# Vascular complications in patients who underwent endovascular cardiac procedures: multicenter cohort study*

**DOI:** 10.1590/1518-8345.2672.3060

**Published:** 2018-10-11

**Authors:** Angelita Costanzi Paganin, Mariur Gomes Beghetto, Maria Karolina Feijó, Roselene Matte, Jaquelini Messer Sauer, Eneida Rejane Rabelo-Silva

**Affiliations:** 1 Unimed, Laboratório de Hemodinâmica, Caxias do Sul, RS, Brazil.; 2 Universidade Federal do Rio Grande do Sul, Escola de Enfermagem, Porto Alegre, RS, Brazil.; 3 Prefeitura Municipal de Porto Alegre, Secretaria Municipal de Saúde, Porto Alegre, RS, Brazil.; 4 Hospital de Clínicas de Porto Alegre, Unidade de Hemodinâmica, Porto Alegre, RS, Brazil.; 5 Instituto de Cardiologia do Rio Grande do Sul, Porto Alegre, RS, Brazil.

**Keywords:** Cardiac Catheterization, Percutaneous Coronary Intervention, Risk Factors, Complications, Postoperative Complications, Nursing Care

## Abstract

**Objective:**

to analyze vascular complications among patients who underwent endovascular cardiac procedures in the hemodynamic laboratories of three referral centers.

**Method:**

a multicenter cohort study was conducted in three referral facilities. The sample was composed of 2,696 adult patients who had undergone elective or urgent percutaneous cardiac procedures. The outcomes were vascular complications, such as: hematoma at the site of the arterial puncture; major or minor bleeding; surgical correction for retroperitoneal hemorrhage; pseudoaneurysm; and arteriovenous fistula.

**Results:**

237 (8.8%) of the 2,696 patients presented a vascular complication at the site of the arterial puncture. The total number of vascular complications was 264: minor hematoma<10cm (n=135); stable bleeding (n=86); major hematoma ≥10cm (n=32); and unstable bleeding (n=11). There were no retroperitoneal hematoma events, pseudoaneurysm or arterial venous fistula. Most of the major and minor complications occurred in the first six hours after the procedure.

**Conclusion:**

the results concerning the current context of interventional cardiology indicate that the complications predominantly occur in the first six hours after the procedure, considering a 48-hour follow-up. The staff should plan and implement preventive measures immediately after the procedures.

## Introduction

The implementation of more complex procedures and aggressive anticoagulation regimes has affected the incidence of complications among patients undergoing endovascular procedures in Hemodynamic Laboratories^1^ ^-^ ^3^. Vascular events stand out among the most frequent complications, such as bleeding at the insertion site, hematoma, pseudoaneurysm, arterial thrombosis, and distal embolization^4^ ^-^ ^7^. 

One study addressed 11,119 patients who had undergone percutaneous coronary intervention (PCI) and 189 (1.7%) of them presented vascular complications. The following predictors of vascular complications were reported: age ≥70 years old (OR=2.4; p<0.001); being a woman (OR=1.6, p<0.001); and body mass index (BMI) (OR=5.8; p<0.05)^8^. Other researchers addressing smaller samples identified rates of 6.5%^1^ and 3.7%^8^ for vascular complications, while one of the studies also considered the use of anticoagulants (OR=3.4, p=0.04), brachial access (OR=3.0, p=0.01), and the long duration of exams (OR=1.4, p<0.001), as being associated with the complications^9^. 

Nevertheless, the rapid development of knowledge and wide availability of a technological arsenal in leading-edge hemodynamics laboratories, combined with the use of more powerful anticoagulation regimens, have impacted the occurrence of vascular complications in patients undergoing invasive cardiac procedures^2^ ^,^ ^10^. 

From this perspective, this multicenter study takes into account this new context in which it is important to update knowledge concerning the incidence of vascular complications, in three referral facilities. This study’s objective is to analyze vascular complications among patients undergoing endovascular cardiac procedures in the hemodynamics laboratories of the three referral centers.

## Methods

This multicenter, prospective cohort study was conducted in three referral centers for Hemodynamics Laboratories in the south of Brazil from October 2013 to March 2014. Two are public university facilities: one has an operational capacity of 845 beds and performs approximately 280 procedures/month in the hemodynamics sector, and the other has 240 beds for hospitalization and 1,000 hemodynamics procedures/month. The third is a private facility located in the second largest city of Rio Grande do Sul, with 112 beds and approximately 110 procedures/month in the Hemodynamics Laboratory.

Patients of both sexes aged ≥18 years old who underwent elective or urgent endovascular procedures (cardiac catheter or PCI) through puncture of the femoral or radial artery were included. Patients lacking the clinical or mental conditions to sign free and informed consent forms or without the presence of a family companion were excluded.

Predictors of complications were identified in previous studies^1^ ^,^ ^8^ ^-^ ^9^ ^,^ ^11^ ^-^ ^14^. A convenience sample, estimated at 3,000 participants, was used. Of these, two thirds composed the derivation cohort and one third composed the validation cohort. Fletcher’s^15^ ^)^ recommendation of including 10 outcomes for each variable kept in the multivariate model was taken into account in the computation. Thus, for the derivation cohort to have up to eight variables in the model, considering an incidence of 3.9% vascular complications in the three facilities (unpublished data), there would be about 2,000 participants necessary. A larger incidence of complications was identified using a preliminary analysis (interim analysis) and we opted to decrease the number of individuals included without losing sample power. 

The staff received training in order to standardize (1) approaching the participants; (2) obtaining the participants’ signature on the free and informed consent forms; (3) the dynamics of data collection; (4) assessment and follow-up of outcomes; and (5) the recording of data on the study’s forms. The research assistants, who were four undergraduate nursing students, were supervised by the head nurses of each of the facilities. 

The patients were initially assessed and reassessed at the time of hospital discharge (or up to 48 hours after), either in person or by analyzing their medical files. There was no follow-up after hospital discharge. A manual containing the operational definitions of each study variable was developed. 

The following outcomes were considered: 1) hematoma at the site of arterial puncture, classified according to the American College of Cardiology (ACC) classifications, large ≥10 cm and small <10 cm^8^; 2) major bleeding, according to criteria presented by the *CRUSADE* ^16^ study, defined as: documented retroperitoneal hemorrhage (without surgical correction) and any transfusion of red blood cells, with bleeding. Also, major bleeding included those with hemodynamics instability defined by uncontrolled hypertension or hypotension, tachycardia or bradycardia or decreased oxygen saturation based on previous baseline parameters, while minor bleeding included the remaining cases, without hemodynamic instability; and 3) surgical correction for any of the vascular complications of retroperitoneal hemorrhage, pseudoaneurysm or arteriovenous fistula formation.

Data were analyzed using the Statistical Package for the Social Sciences (SPSS) v.22. Descriptive analysis was initially performed. Continuous variables were expressed through mean and standard deviation or median (interquartile interval), according to distribution and the categorical variables were expressed in percentages and absolute numbers.

The incidence of each of the outcomes was calculated, in addition to grouping them into vascular complications or other complications. To identify the incidence of complications according to the period in which the occurrence took place, the time elapsed up to the development of complications was categorized as: (1) between zero hour and 6^th^ incomplete hour; (b) between the 6^th^ hour and 24^th^ incomplete hour; and (c) between the 24^th^ hour and 48 hours after the procedure. P-values<0.05 (two-tailed) were considered statistically significant. 

This study was approved by the Institutional Review Boards at each of the facilities (HCPA 120,469, IC-FUC 114,772) and by the Unimed Hospital Management Board as recommended by resolution 466/12, which regulates research involving human subjects. All the researchers signed a document regulating the use of information collected from the patients’ files. 

## Results

Out of a total of 2,718 potentially eligible patients, 22 were excluded: 13 for refusing to participate and nine for presenting mental confusion or hemodynamic instability at the time of data collection, lacking the presence of a family companion, so that 2,696 patients remained. 

Average age was 63±11 years old and males predominated with 60%. Comorbidities, such as systemic blood pressure (SBP), Dyslipidemia, and Diabetes Mellitus (DM), were most frequently found. The sample’s characteristics are presented in Table 1.

**Table 1:** Characteristics of the sample (n=2,696) of patients who underwent endovascular cardiac procedures. Caxias do Sul and Porto Alegre, RS, Brazil. 2012-2014

Variables	n (%)
Age (years)*	63±11
Sex (male)	1612 (59.8)
Cardiac catheterization diagnosis	2023 (75)
Systemic blood pressure	2281 (84.6)
Dyslipidemia	79 (72.5)
Diabetes mellitus	816 (30.3)
Kidney failure	92 (3.4)
Dialysis method	31 (1.2)
Prior hemodynamic procedure	1135 (42.1)
Prior hemodynamic vascular complication	289 (10.7)
Prior peripheral arterial disease	271 (10.1)
Prior anticoagulation	1992 (73.9)

*Variables expressed with mean ± standard deviation

**Figure 1:**
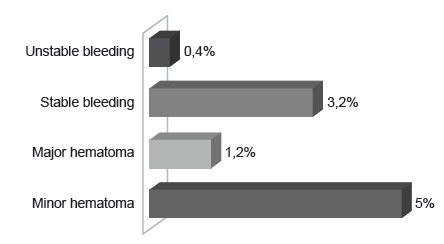
Incidence of vascular complications according to type of event

A total of 237, out of 2,696 patients, presented some type of vascular complication (8.8%). The following results refer to vascular complications analyzed according to event, considering that patients may have experienced more than one complication. The total number of complications was 264 (9.8%), distributed as follows: minor hematoma <10cm (n=135), followed by stable bleeding (n=86), major hematoma ≥10cm (n=32), and unstable bleeding (n=11). No retroperitoneal hematoma, pseudoaneurysm, or arteriovenous fistula occurred. Data presented in Figure 1.

Table 2 shows the incidence of complications at three different points in time: between zero hour and 6^th^ incomplete hour; between the 6^th^ hour and 24^th^ incomplete hour; and between the 24^th^ hour and 48^th^ hour after the procedure. All the patients (n=2,696) were assessed immediately after the procedure (in the first 6 hours), while 1,349 (50.1%) and 356 (13.2%) were assessed again in the two subsequent periods, respectively. Data show that 97.3% of the major complications and 96.8% of the minor complications occurred in the first six hours after the procedure. There is a difference in the number of patients for each complication due to missing data.

**Table 2:** Incidence of vascular complications from 0-6h, 6-24h, and 24-48h. Caxias do Sul and Porto Alegre, RS, Brazil. 2012-2014

Vascular complications (n)	0-6h n (%)	6-24h n (%)	24-48h n (%)	Total n (%)
Major complications (n= 2,659)	36 (1.3)	1 (<0.1)	0 (0)	37 (1.4)
Major hematoma (≥ 10cm)(n=2,667)	28 (1)	1 (<0.1)	0 (0)	29 (1.1)
Unstable bleeding (n= 2,688)	8 (0.3)	0 (0)	0 (0)	8 (0.3)
Pseudoaneurysm (n= 2,696)	0 (0)	0 (0)	0 (0)	0 (0)
Retroperitoneal hematoma (n= 2696)	0 (0)	0 (0)	0 (0)	0 (0)
Arteriovenous fistula (n= 2696)	0 (0)	0 (0)	0 (0)	0 (0)
Minor complications (n= 2,506)	184 (6.8)	5 (0.2)	1 (<0.1)	190 (7)
Major hematoma (<10cm)(n= 2,573)	118 (4.4)	4 (0.1)	1 (<0.1)	123 (4.6)
Stable bleeding (n= 2,612)	83 (3.1)	1 (<0.1)	0 (0)	84 (3.1)

“Other complications” were also investigated (vasovagal reaction, allergy, pyrogenic, arrhythmia, ischemia, embolism, and congestive and neurological complications) and 132 (4.9%) complications were found. Table 3 presents the occurrence of “other complications” stratified by subtype. A greater incidence of these complications was also observed in the first six hours after the procedure. There is also a difference in the number of patients according to type of complication due to missing data.

**Table 3:** Incidence of other immediate complications within 24h and 48h. Caxias do Sul and Porto Alegre, RS, Brazil. 2012-2014

Other complications (n)	0-6h n (%)	6-24h n (%)	24-48h n (%)	Total n (%)
Vasovagal (n= 2,627)	68 (2.5)	1 (<01)	0 (0)	69 (26)
Allergy (n= 2,664)	30 (11)	1 (<01)	1 (<01)	32 (12)
Pyrogenic (n= 2,682)	13 (05)	1 (<01)	0 (0)	14 (05)
Arrhythmia (n= 2,691)	3 (01)	2 (01)	0 (0)	5 (02)
Ischemia (n= 2,692)	1 (<01)	2 (01)	1 (<01)	4 (01)
Embolism (n= 2,694)	1 (<01)	0 (0)	1 (<01)	2 (01)
Congestive (n= 2,693)	2 (01)	0 (0)	1 (<01)	3 (01)
Neurological (n= 2,693)	2 (01)	0 (0)	1 (<01)	3 (01)

Two (0.1%) of the patients died, while one of the cases was potentially related to embolic complication. 

## Discussion

This study presents the results of a multicenter study addressing the incidence of vascular complications in the current context of interventionist cardiology. Considering all the complications addressed here (major and minor vascular complications), the percentage remained below 10%. Some studies do not address minor complications, so that, if we verify the incidence of major complications only, here defined as hematoma ≥10cm, unstable bleeding, retroperitoneal hematoma, pseudoaneurysm, and arteriovenous fistula, the rate remains at 1.6%, including diagnostic and therapeutic procedures. A rate of 1.6% is relatively low, when compared to results previously reported that considered only major complications, at approximately 3%^1^ ^,^ ^17^. 

One study addressing 194,476 cardiac catheterizations and 85,024 PCI procedures performed in hemodynamic laboratories, was recently published in the United States, showing that patients undergoing these procedures were progressively older, presented more comorbidities, and that their medical management after the procedures remained unchanged over the period, though there was an increasing adoption of transradial access for diagnostic procedures (from 6% to 36%; p <0.001) and interventions (from 5% to 32%; p <0.001). Complications and clinical outcomes also remained constant, with a downward tendency^18^. The study addressed patients whose procedures included arterial femoral and transradial access. Attention, however, should be paid to complications, regardless of the type of access chosen, because even though transradial access has been increasingly used, many procedures are still performed using the femoral route. Thus, patients need to be continuously assessed by the nursing staff to rapidly identify events. 

Another study intending to decrease the rate of vascular complications after procedures performed in the femoral artery reports that the use of fluoroscopic demarcation of the femoral head before access, small-sized introducers, and implementing the procedure in a referral center, contribute to decreasing the incidence of vascular complications^19^. The variables identified as protective factors for the non-occurrence of vascular complications are relevant, as the staff can opt to use small caliber introducers, which in fact decreases vascular complications^20^. 

Vascular complications most frequently took place in the first six hours after the invasive procedure, showing that the nursing staff has the opportunity to take action in order to prevent and decrease the frequency of major complications. Patients in recovery require special attention, that is, individual and integral care^21^ ^-^ ^23^. Nursing prescriptions in the postoperative period should include the duration of rest, verification of pulse, site of puncture, vital signs, and emphasize care concerning bleeding and hematoma. The nursing staff should be qualified to safely implement care.

When the incidence of other complications is analyzed, vasovagal reaction appears as the most frequent complication, at 2.6%, a finding that is also reported by other studies^9^. A study recently conducted to predict the risk of vasovagal reaction among patients who underwent PCI, reports an incidence of 4.5%, the independent factors included being a woman, primary coronary angioplasty, SBP, more than two stents implanted in the anterior descending artery, and puncture at the femoral site^24^. When patients who underwent cerebral angiography exclusively using the femoral access are included, indexes vary little (4.09%)^25^. Despite its low incidence, when compared to other complications, vasovagal reaction should not be underestimated, given the risk of cardiorespiratory arrest. Thus, it should be addressed in training programs and be constantly supervised by the nursing staff.

Similar to previous studies, allergy was not a very frequent event^9^ ^,^ ^26^ ^-^ ^27^. Hypersensitive responses should be taken into account when choosing the contrast media for procedures. A double-blind randomized study assessed the nonionic contrast, iso-osmolar, and low-osmolarity ionic contrast and verified that hypersensitive responses (2.5% *vs.* 0.7%) were statistically less frequent (p=0.007) in the group using the nonionic and iso-osmolar contrast media^28^. Due to the use of increasingly modern contrast media with low osmolarity, allergic responses are increasingly rare, so that patient safety has advanced in this aspect. 

Finally, the results found in this multicenter cohort study indicate that nurses from hemodynamic laboratories should be attentive to risk factors such as the caliber of introducer used, the prior use of anticoagulation, prior vascular complications, advanced age, being a woman, and percutaneous coronary intervention. Well-planned interventions implemented in the first six hours can change the course of patient care, improving the safety and quality of care. 

Limitations for this study include the fact that procedures other than cardiac procedures performed in hemodynamics were not included.

This study’s findings bring important contributions to the clinical practice of nursing staff; that is, nursing staff needs to be aware of the complications and risk factors in order to develop more efficacious care actions for their patients.

## Conclusion

The results show that the general incidence of (major and minor) vascular complications in the first 48 hours in three referral centers in the south of Brazil is lower than that reported in many international referral centers. There was no occurrence of pseudoaneurysm, retroperitoneal hematoma, or arteriovenous fistula in this study. In regard to other complications, those with the highest incidence were vasovagal reactions and allergic responses.

The incidence of these complications predominantly occurred in the first six hours after the procedures, considering a 48-hour follow-up. The staff should plan preventive measures to be implemented immediately after procedures.

This study’s findings contribute to knowledge concerning complications that patients undergoing endovascular cardiac procedures may experience, which can support the planning of care provided before and after procedures.

## References

[1] Brito FS, Magalhães MA, Nascimento TCDC, Amorim IMG, Almeida BO, Abizaid A, ET AL (2007). Incidence and contemporary predictors of vascular complications
after percutaneous coronary interventions. Rev Bras Cardiol Invasiva.

[2] Yang E, Ipek EG, Balouch M, Mints Y, Chrispin J, Marine JE (2017). Factors impacting complication rates for catheter ablation of
atrial fibrillation from 2003 to 2015. Europace.

[3] Steg PG, James S, Harrington RA, Ardissino D, Becker RC, Cannon CP (2010). Ticagrelor Versus Clopidogrel in patients with ST-elevation acute
coronary syndromes intended for reperfusion with primary percutaneous
coronary intervention a Platelet Inhibition and Patient Outcomes (PLATO)
trial subgroup analysis. Circulation.

[4] Armendaris MK, Azzolin KO, Alves FJMS, Ritter SG, Moraes MAP (2008). Incidence of vascular complications in patients submitted to
percutaneous transluminal coronary angioplasty by transradial and
transfemoral arterial approach. Acta Paul Enferm.

[5] Lima LR, Stival MM, Lima LR (2008). Nursing diagnoses in patients post-angioplasty transluminal
percutaneous coronary based on the Horta's assumption. Rev Enferm UFPE On Line.

[6] Sedlacek MA, Newsome J (2010). Identification of vascular bleeding complications after cardiac
catheterization through development and implementation of a cardiac
catheterization risk predictor tool. Dimens Crit Care Nurs.

[7] Andrade PB, Andrade MVA, Barbosa RA, Labrunie A, Hernandes ME, Marino RL (2014). Femoral versus Radial Access in Primary Angioplasty Analysis of
the ACCEPT Registry. Arq Bras Cardiol.

[8] Dumont CJP, Keeling AW (2006). Bourguignon C, Sarembock IJ, Turner M Predictors of vascular
complications post diagnostic cardiac catheterization and percutaneous
coronary interventions. Dimens Crit Care Nurs.

[9] Rossato G, Quadros AS, Sarmento-Leite R, Gottschall CAM (2007). Analysis of in-hospital complications related to cardiac
catheterization. Rev Bras Cardiol Invas.

[10] Van Mieghem NM, Latib A, van der Heyden J, van Gils L, Daemen J, Sorzano T (2017). Percutaneous Plug-Based Arteriotomy Closure Device for Large-Bore
Access A Multicenter Prospective Study. JACC Cardiovasc Interv.

[11] Qureshi MA, Safian RD, Grines CL, Goldstein JA, Westveer DC, Glazier S (2003). Simplified scoring system for mredicting Mortality after
percutaneous coronary intervention. J Am Coll Cardiol.

[12] Singh M, Peterson ED, Milford-Beland S, Rumsfeld JS, Spertus JA (2008). Validation of the Mayo Clinic Risk Score for in-hospital
mortality after percutaneos coronary interventions using the national
cardiovascular data registry. Circ Cardiovasc Interv.

[13] Ahmed B, Liscke S, De Sarno M, Holterman LA, Straight F, Dauerman HL (2013). Gender related differences in predictors of vascular
complications role of vessel and BMI. J Thromb Thrombolysis.

[14] Shin JS, Tahk SJ, Yang HM, Yoon MH, Choi SY, Choi BJ (2014). Impact of female gender on bleeding complications after
transradial coronary intervention (from the Korean Transradial Coronary
Intervention registry). Am J Cardiol.

[15] Fletcher RH, Frisancho AR, Wagner EH, Fletcher RH (1996). Chance. Clinical epidemiology the essentials.

[16] Subherwal S, Bach RG, Chen AY, Gage BF, Rao SV, Newby CV (2009). Baseline risk of major bleeding in non-ST-segment-elevation
myocardial infarction the CRUSADE (Can Rapid risk stratification of Unstable
angina patients Suppress Adverse outcomes with Early implementation of the
ACC/AHA guidelines) Bleeding Score. Circulation.

[17] Zanatta LG, Cardoso CO, Mota FM, Conti EP, Diehl D, Rodrigues APR (2008). Predictors and incidence of vascular complications after
percutaneous coronary interventions findings from the IC-FUC
Registry. Rev Bras Cardiol Invas.

[18] Waldo SW, Gokhale M, O´Donnell Cl, Plomondon ME, Valle JA, Armstrong EJ (2018). Temporal Trends in Coronary Angiography and Percutaneous Coronary
Intervention Insights From the VA Clinical Assessment, Reporting, and
Tracking Program. JACC Cardiovasc Interv.

[19] Bogabathina H, Shi R, Singireddy S, Morris L, Abdulbaki A, Zabher H (2018). Reduction of vascular complication rates from femoral artery
access in contemporary women undergoing cardiac
catheterization. Cardiovasc Revasc Med.

[20] Paganin AC, Beghetto MG, Hirakata VN, Hilário TS, Matte R, Sauer JM (2017). A Vascular Complications Risk (VASCOR) score for patients
undergoing invasive cardiac procedures in the catheterization laboratory
setting A prospective cohort study. Eur J Cardiovasc Nurs.

[21] Rocha VS, Aliti G, Moraes MA, Rabelo ER (2009). Three-hour rest period after cardiac catheterization with a 6 F
sheath does not increase complications a randomized clinical
trial. Rev Bras Cardiol Invas.

[22] Matte R, Hilário TS, Reich R, Aliti GB, Rabelo-Silva ER (2016). Reducing bed rest time from five to three hours does not increase
complications after cardiac catheterization the THREE CATH Trial. Rev.
Latino- Am. Enfermagem.

[23] Paganin A, Rabelo ER (2013). Clinical Validation of the nursing diagnoses of Impaired Tissue
Integrity and Impaired Skin Integrity in patients subjected to cardiac
catheterization. J Adv Nurs.

[24] Li HY, Guo YT, Tian C, Song CQ, Mu Y, Li Y (2017). A risk prediction score model for predicting occurrence of
post-PCI vasovagal reflex syndrome a single center study in Chinese
population. J Geriatr Cardiol.

[25] Yang Y, Zhang Z, Li T, Gu Z, Sun Y (2017). Risk factors for vasovagal reaction associated with cerebral
angiography via femoral catheterisation. Interv Neuroradiol.

[26] Nunes GL, Nicolela EL, Sousa GM, Maldonado G, Cano MM, Esteves CA (1991). Current complications of heart catheterization: analysis of 1000
cases. Arq Bras Cardiol.

[27] Noto TJ, Johnson LW, Krone R, Weaver WF, Clark DA, Kramer JR (1991). Cardiac catheterization 1990: a report of the registry of Society
of Cardiac Angiography and Interventions (SCA&I). Cathet Cardiovasc Diagn.

[28] Bertrand ME, Esplugas E, Piessens J, Rasch W (2000). Influence of a Nonionic, Iso-Osmolar Contrast Medium (Iodixanol)
Versus an Ionic, Low-Osmolar Contrast Medium (Ioxaglate) on Major Adverse
Cardiac Events in Patients Undergoing Percutaneous Transluminal Coronary
Angioplasty. Circulation.

